# Combination Treatment of Rosuvastatin or Atorvastatin, with Regular Exercise Improves Arterial Wall Stiffness in Patients with Coronary Artery Disease

**DOI:** 10.1371/journal.pone.0041369

**Published:** 2012-07-19

**Authors:** Kensuke Toyama, Seigo Sugiyama, Hideki Oka, Yuri Iwasaki, Hitoshi Sumida, Tomoko Tanaka, Shinji Tayama, Hideaki Jinnouchi, Hisao Ogawa

**Affiliations:** 1 Faculty of Life Science, Department of Cardiovascular Medicine, Graduate School of Medical Sciences, Kumamoto University, Kumamoto, Japan; 2 Research Fellowship of the Japan Society for the Promotion of Science, Tokyo, Japan; 3 Division of Cardiology, Health Insurance Hitoyoshi General Hospital, Kumamoto, Japan; 4 Clinical Research Center, Health Insurance Hitoyoshi General Hospital, Kumamoto, Japan; 5 Division of Cardiology, Kumamoto Central Hospital, Kumamoto, Japan; 6 Division of Preventive Cardiology, Department of Cardiovascular Medicine, Kumamoto University Hospital, Kumamoto, Japan; Brigham and Women's Hospital, Harvard Medical School, United States of America

## Abstract

**Objective:**

Statin- and exercise-therapy are both clinically beneficial by preventing cardiovascular events in patients with coronary artery disease (CAD). However, there is no information on the vascular effects of the combination of statins and exercise on arterial wall stiffness in CAD patients.

**Methods:**

The present study is a sub-analysis of PRESET study that determined the effects of 20-week treatment with statins (rosuvastatin, n = 14, atorvastatin, n = 14) combined with regular exercise on arterial wall stiffness assessed by measurement of brachial and ankle pulse wave velocity (baPWV) in CAD patients.

**Results:**

The combination of statins and regular exercise significantly improved exercise capacity, lipid profile, including low- and high-density lipoprotein cholesterol, and high-sensitivity C-reactive protein (hs-CRP), baPWV (baseline: 1747±355, at 20 weeks of treatment: 1627±271 cm/s, p = 0.008), and basophil count (baseline: 42±32, 20 weeks: 26±15 cells/µL, p = 0.007), but had no effect on blood pressure (baseline: 125±22, 20 weeks: 121±16 mmHg). Changes in baPWV correlated significantly with changes in basophil count (r = 0.488, p = 0.008), but not with age, lipids profile, exercise capacity, or hs-CRP.

**Conclusion:**

In CAD patients, the combination treatment with statins and exercise resulted in significant amelioration of arterial wall stiffness, at least in part, through reduction of circulating basophils.

## Introduction

The importance of exercise for patients with coronary artery disease (CAD) has been demonstrated [1], [2]. Furthermore, the use of 3-hydroxy-3-methylglutaryl coenzyme A reductase (HMG-CoA reductase; statin) is effective in reducing cardiovascular events [3]. Thus, the combination of statin and regular exercise should improve quality of life and prognosis, and should be recommended for all CAD patients. We reported previously in the study of possible role of statin treatment on exercise training (PRESET study) investigating the superiority of rosuvastatin, compared to atorvastatin, combined with exercise in increasing serum high-density lipoprotein cholesterol (HDL-C) [4].

Inflammation of the arterial wall is associated with increased arterial wall stiffness and the latter, which is assessed clinically by measurement of pulse wave velocity (PWV), is associated with increased risk for cardiovascular events [5],[6]. Reduction of the PWV is considered a potentially useful therapeutic strategy in the overall management of patients with cardiovascular disease. However, there is little or no information on the synergistic effects of the combination of statin treatment and exercise on arterial wall stiffness and inflammatory markers in patients with CAD. The aim of the present study was to determine the effects of statin therapy (rosuvastatin or atorvastatin) combined with regular exercise on arterial wall stiffness, and to clarify the relationship between inflammatory markers and arterial wall stiffness in patients with CAD.

## Materials and Methods

### Study Design

The design of this study (identification number UMIN000002799) has already been described in detail [4]. After dividing 28 Japanese CAD patients with a history of myocardial infarction, angina pectoris or 50% or more stenosis in at least one major coronary artery into an atorvastatin (n = 14) and a rosuvastatin (n = 14) group at random, the patients performed weekly in-hospital aerobic training for half an hour on a bicycle ergometer based on their anaerobic threshold for 20 weeks. A well-trained registered exercise rehabilitation instructor instructed all patients on carrying out home training for half an hour by walking with Borg Scale 12–13 during the study. Atorvastatin was administered at doses from 10 to 40 mg/day and rosuvastatin from 2.5 to 20 mg/day, without any other lipid-lowering drug, to lower low-density lipoprotein cholesterol (LDL-C) levels below 100 mg/dL, according to the guidelines of the Japan Atherosclerosis Society [7]. The protocol of the PRESET study was approved by the Institutional Review Board of Health Insurance Hitoyoshi General Hospital in Kumamoto, Japan and a signed consent form was obtained from each subject.

### Measurement of Brachial and Ankle Pulse Wave Velocity

The patient was examined in the morning following an overnight fast at the time of enrollment and at end of the study. The brachial and ankle PWV (baPWV) was measured after a 10 min-rest using an automatic waveform analyzer (form PWV/ABI, Colin, Komaki, Japan) while the subject was in the supine position. As described previously [8], we analyzed the higher baPWV level measured on either side of each patient at the beginning of the study, and repeated the analysis on the same side in each patient during the study.

### Leucocytes Count and High-Sensitivity C-Reactive Protein (hs-CRP) Assay

As reported previously [4], venous blood samples were collected in the morning before the exercise test following an overnight fast. Total leukocyte and differential leukocyte counts were determined automatically using a XE-2100 (Sysmex Corporation, Japan). The hs-CRP level was measured by nephelometry with BNII (Siemens, Berlin, Germany) at the Japan SRL Laboratory (Tokyo).

### Statistical Analysis

The present study is a sub-analysis of the PRESET study which has been conducted to investigate the different effects of atorvastatin or rosuvastatin combined with regular exercise on the serum ubiquinol levels. As mentioned previously [4], power analysis indicated that an enrollment of 22 patients was required to detect a mean decrease in ubiquinol levels of 350 nmol/L in atorvastatin group and 0 nmol/L in rosuvastatin group, with a power of 80% and a two-sided alpha of 0.05.

To compare the baseline characteristics of the two groups, data were analyzed by the Student's *t*-test or Mann-Whitney U test, as appropriate. A paired Student's *t*-test or Wilcoxon test was performed to analyze the effect of 20-week treatment. The Pearson correlation test was used to analyze the correlation between changes in baPWV and other parameters. Hazard ratios, 95% confidence intervals (95%CI), and levels of statistical significance (p values) were calculated. Data were expressed as mean ± standard deviation (SD) or median values, and p<0.05 was considered statistically significant. All statistical analyses were performed using the Statistical Package for Social Sciences, version 11.0J (SPSS Inc., Chicago, IL).

## Results

### Patient Characteristics

**Figure 1:**
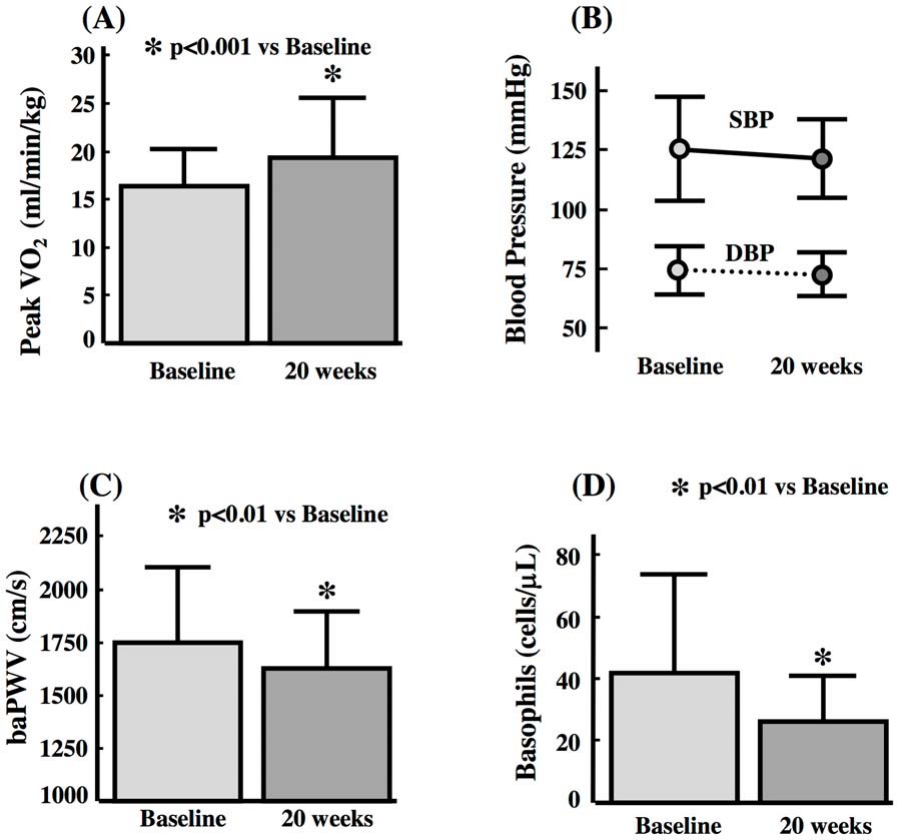
Effect of Combination Treatment on Arterial Wall Stiffness and Plasma Basophil. Effects of treatment on (A) peak oxygen uptake (peak VO_2_), (B) blood pressure, (C) arterial wall stiffness measured by brachial and ankle pulse wave velocity (baPWV) and (D) plasma basophil count using data of all patients (n = 28). Data are mean±SD. SBP, systolic blood pressure; DBP, diastolic blood pressure.

Table 1 summarizes the baseline characteristics of the patients in the PRESET study. As mentioned previously [4], cardiopulmonary function measured by peak oxygen uptake (Peak VO_2_) significantly improved after the combination treatment of statins plus regular exercise in the entire group (Figure 1A).

**Table 1:** Patients' Characteristics.

n	28
Age (years)	67±11
Male Gender, n (%)	21 (75%)
Body Mass Index (kg/m^2^)	24.5±3.0
Left Ventricular Ejection Fraction (%)	60.5±7.9
E/A Ratio	0.9±0.3
Hypertension, n (%)	25 (89%)
Diabetes, n (%)	14 (50%)
Dyslipidemia, n (%)	23 (82%)
Smoking, n (%)	8 (29%)
Myocardial Infarction, n (%)	9 (32%)
Angina Pectoris, n (%)	14 (50%)
Multi Vessel Disease, n (%)	14 (50%)
Aspirin, n (%)	26 (93%)
Angiotensin Converting Enzyme Inhibitors, n (%)	10 (36%)
Angiotensin II Receptor Blockers, n (%)	14 (50%)
Calcium Channel Blockers, n (%)	14 (50%)

Data are mean ± SD or percentage of patients.

### Changes in Blood Pressure and Arterial Wall Stiffness

Systolic and diastolic blood pressure in all patients did not change significantly during the study (125±22 mm Hg to 121±16 mm Hg, p = 0.227, Figure 1B). Mean arterial pressures did not change either after treatment (91±12 mm Hg to 89±10 mm Hg, p = 0.329). As shown in Figure 1C, the combination treatment significantly reduced the baPWV of the entire group. Changes in baPWV did not correlate significantly with changes in mean arterial pressures (r = 0.245, p = 0.209).

### Changes in Inflammatory Factors, Metabolic Parameters, and Ubiquinol

The combination treatment significantly reduced plasma basophil count and serum hs-CRP level (Figure 1D, Table 2), significantly increased HDL-C and significantly reduced total cholesterol (T-Cho), triglyceride, LDL-C, non-HDL, and ubiquinol for the whole group (Table 2). However, the same treatment had no effect on total leukocytes, differential leukocyte counts; neutrophil, lymphocyte, monocyte, eosinophil counts, fasting blood glucose and hemoglobin A1c (HbA1c) during the study (Table 2).

**Table 2:** Effects of Statin-Exercise Treatment on Various Parameters.

All (n = 28)	Baseline	20 weeks	p value
Total Cholesterol (mg/dL)	202±35	147±24	<0.001
High-Density Lipoprotein Cholesterol (mg/dL)	46±14	54±14	<0.001
Triglyceride (mg/dL)	133±47	90±42	<0.001
Low-Density Lipoprotein Cholesterol (mg/dL)	129±31	75±14	<0.001
Non-High-Density Lipoprotein Cholesterol (mg/dL)	156±35	93±18	<0.001
Ubiquinol (nmol/L)	706±233	608±264	0.016
Fasting Blood Glucose (mg/dL)	104±19	101±15	0.324
Hemoglobin A1c (%)	5.8±0.8	5.6±0.6	0.231
Total Leukocyte Count (cells/µl)	5225±1472	4980±1263	0.286
- Neutrophil (cells/µl)	3217±1048	2968±1007	0.211
- Lymphocyte (cells/µl)	1465±449	1511±439	0.465
- Monocyte (cells/µl)	303±116	278±102	0.144
- Eosinophil (cells/µl)	199±107	198±151	0.940
High-Sensitivity C-Reactive Protein (ng/mL)	1430 (264–2478)	307 (146–623)	<0.001

Data are mean ± SD or median values (25^th^ to 75^th^ percentile range).

### Relationship Between Arterial Wall Stiffness and Inflammatory Factors

**Figure 2:**
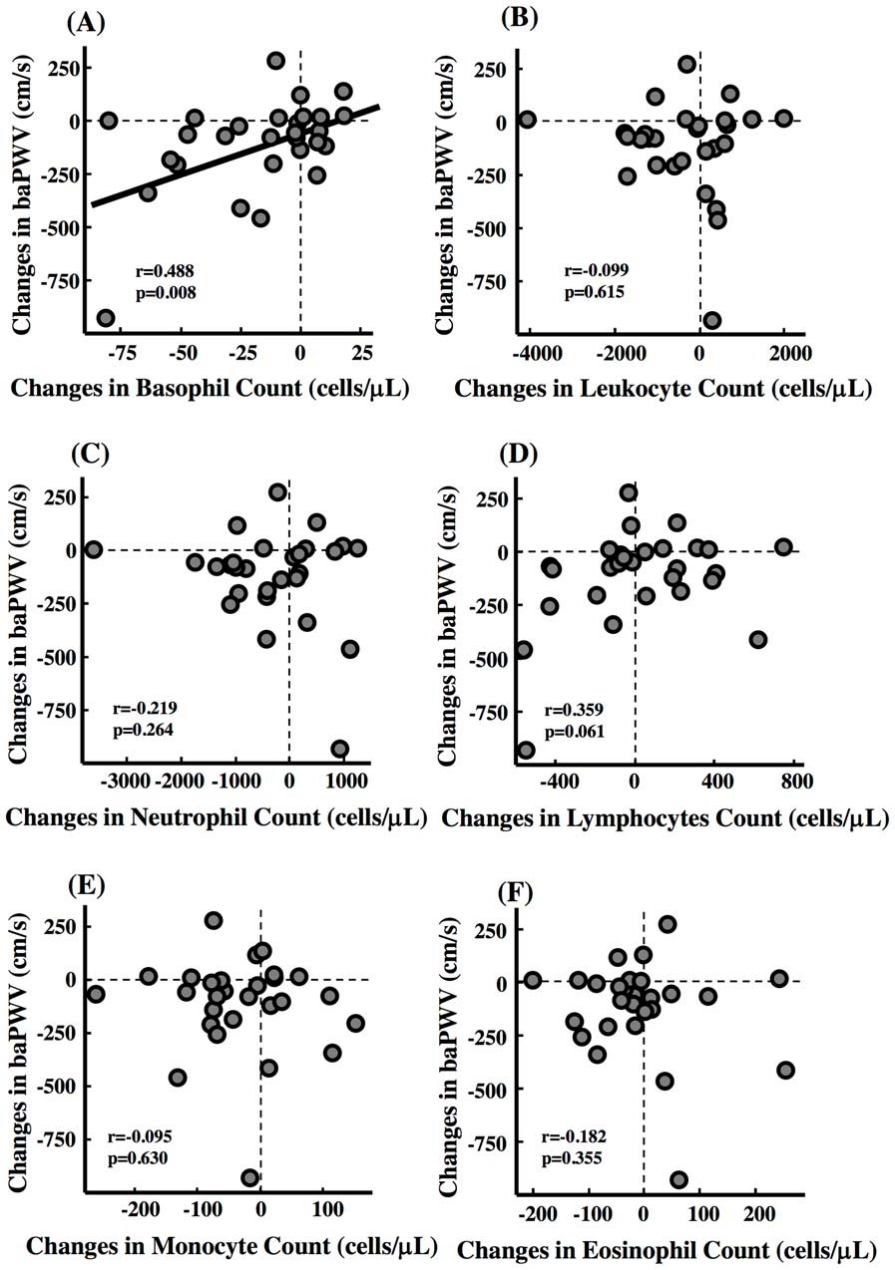
Association Between Arterial Wall Stiffness and Each Plasma Leukocyte. Correlations between changes in brachial and ankle pulse wave velocity (baPWV) and (A) basophil count, (B) total leukocyte count, (C) neutrophil count, (D) lymphocyte count, (E) monocyte count, (F) eosinophil count.

**Figure 3:**
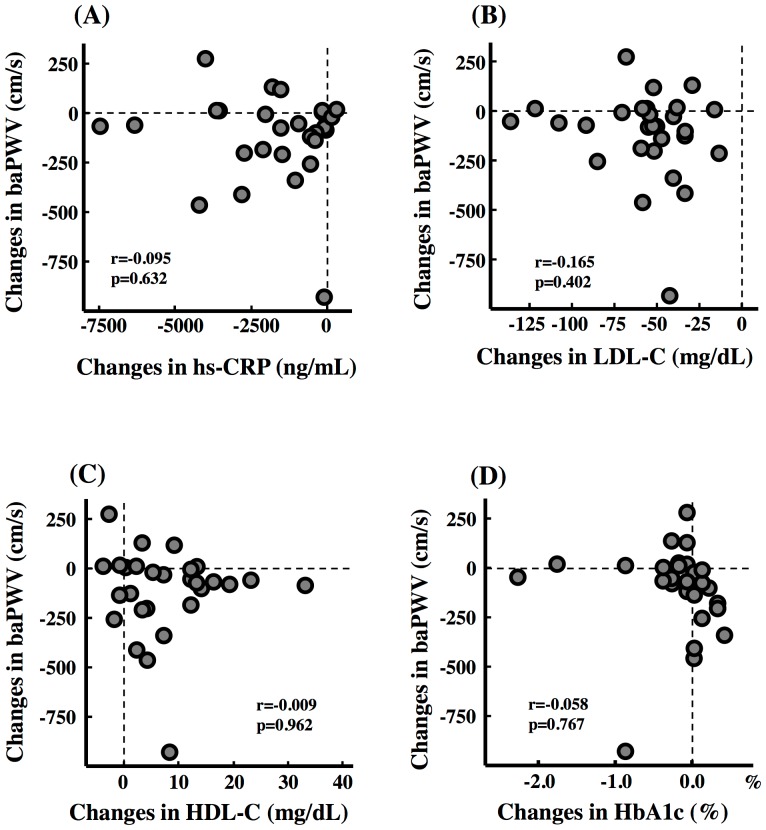
Association Between Arterial Wall Stiffness and Other Parameters. Correlations between changes in brachial and ankle pulse wave velocity (baPWV) and (A) high-sensitivity c-reactive protein (hs-CRP), (B) low-density lipoprotein cholesterol (LDL-C), (C) high-density lipoprotein cholesterol (HDL-C) and (D) hemoglobin A1c (HbA1c).

Changes in basophil count correlated significantly with the changes in baPWV in all patients (Figure 2A). There were not significant correlations between changes in total leukocytes, neutrophil, lymphocyte, monocyte, eosinophil, and hs-CRP and changes in baPWV (Figures 2B–F and 3A).

### Relationship Between Arterial Wall Stiffness and Other Parameters

Changes in baPWV did not correlate significantly with age, changes in Peak VO_2_, T-Cho, triglyceride, LDL-C, HDL-C, non-HDL, ubiquinol, fasting blood glucose and HbA1c (Figure 3B–D, Table 3).

**Table 3:** Correlations Between Changes in PWV and Other Parameters.

	r	p value
Age (years)	−0.060	0.763
Peak Oxygen Uptake	0.270	0.165
Total Cholesterol (mg/mL)	−0.099	0.616
Triglyceride (mg/mL)	0.214	0.274
Non-High-Density Lipoprotein Cholesterol (mg/mL)	−0.101	0.608
Ubiquinol (nmol/L)	−0.236	0.226
Fasting Blood Glucose (mg/dL)	−0.085	0.669

### Comparison of Arterial Wall Stiffness and Change in Plasma Basophil in Rosuvastatin and Atorvastatin Groups

**Figure 4:**
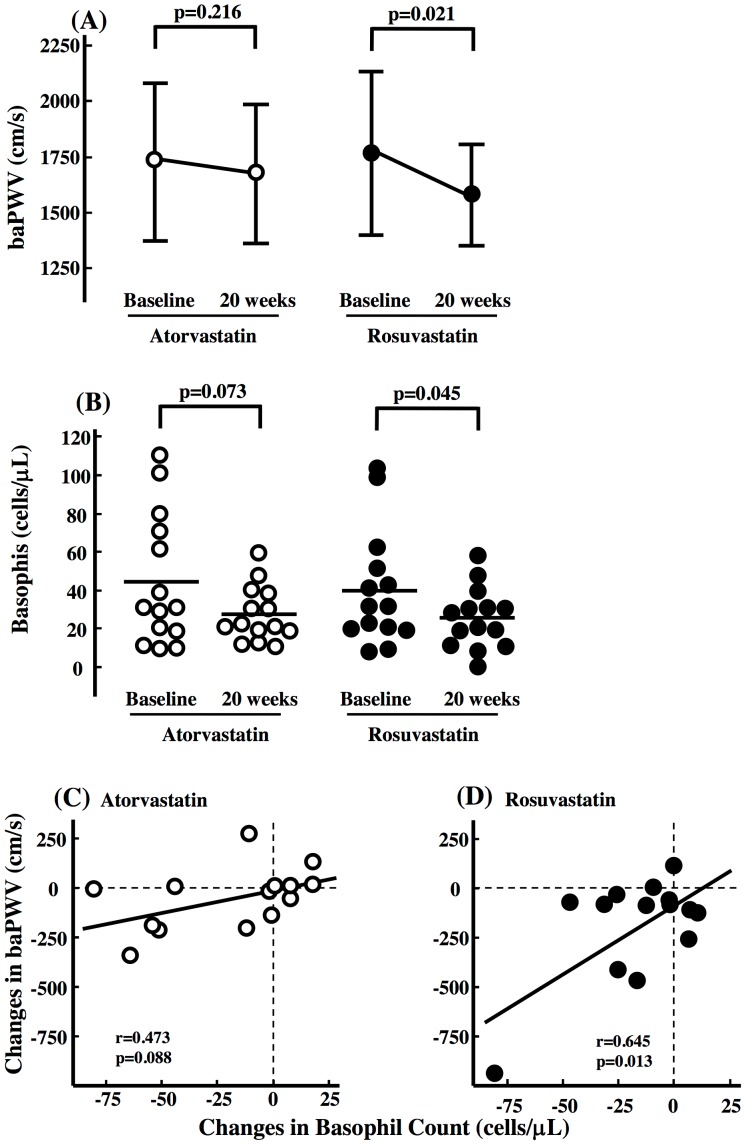
Effect of Atorvastatin or Rosuvastatin Combined with Exercise on Arterial Wall Stiffness and Plasma Basophil. Comparison of changes in (A) brachial and ankle pulse wave velocity (baPWV) and (B) basophil count, between atorvastatin (n = 14) and rosuvastatin (n = 14) groups. Data are mean±SD. Correlation between changes in baPWV and basophil count in (C) atorvastatin and (D) rosuvastatin groups.

Finally, the test parameters were analyzed according to the type of statin used for treatment. The baseline baPWV (atorvastatin: 1729±355, rosuvastatin: 1766±367 cm/s, p = 0.789) and plasma basophil count (atorvastatin: 44±34, rosuvastatin: 40±30 cells/µL, p = 0.703) were not significantly different between the two groups. Rosuvastatin combined with regular exercise significantly decreased baPWV (Figure 4A) and basophil count (Figure 4B), but atorvastatin did not. Changes in baPWV correlated significantly with plasma basophil count in the rosuvastatin group (Figure 4D), but not in the atorvastatin group (Figure 4C).

## Discussion

The major finding of our study is that the combination of statins (atorvastatin or rosuvastatin) and regular exercise significantly ameliorated arterial wall stiffness, which correlated with reduced basophil count in patients with CAD.

Increased arterial wall stiffness is a risk factor for cardiovascular events through increased cardiac afterload, impaired coronary blood flow, atherogenesis and microangiopathies [5]. Several methods, such as flow-mediated vasodilation and PWV, are used to assess vascular function. PWV is a useful method to assess increased arterial wall stiffness. Reduction in PWV levels is potentially beneficial in the management of patients with CAD. Advanced age, glucose intolerance, lipid profile abnormalities, high CRP, and oxidative stress correlate with increased arterial wall stiffness [9]–[12], however we could not find in the present study any significant association between the above factors and changes in arterial wall stiffness assessed by PWV. Aerobic exercise in patients with CAD plays a beneficial role in secondary prevention by improvement of cardiopulmonary function, modulation of various cardiometabolic risk factors [1], [13], and amelioration of arterial wall stiffness [14]. Statins, such as pravastatin, have been reported to improve arterial wall stiffness in association with a reduction in leukocyte subtypes such as monocytes [15]. However, the effect of statins on arterial wall stiffness was not confirmed in a recent meta-analysis study [16]. Our study confirmed the clinical effectiveness of the combination therapy of statin and exercise in reducing increased arterial wall stiffness in CAD patients.

Basophils represent less than 1% of the total leukocyte count in peripheral blood and are considered possibly redundant relative to mast cells in tissues. New information on their function and clinical significance has emerged recently. T helper type 1 (Th1) lymphocytes produce interferon-γ, interleukin (IL)-2 and tumor necrosis factor-α, which are associated with cell-mediated immunity, while T helper type 2 (Th2) lymphocytes produce IL-4, 5 and 10, which are associated with humoral immunity and antibody production. Recent studies have reported the importance of basophils in the initiation of Th2 responses by secreting IL-4, which is involved in the development of immunological abnormalities, such as allergic and autoimmune disorders [17]. However, further details are needed about the potential involvement of basophils and basophil-mediated immune response in cardiovascular diseases. In the early and mid-1990s, the importance of mast cells, but not basophils, in the pathogenesis of atherosclerosis and vascular diseases was reported [18], [19]. Both the Th1 response and the Th2 response are important in the process of atherosclerogenesis [20]. Furthermore, Schonbeck et al. [21] have shown that Th2 cytokines and Th2 characteristic response predominate in human aortic aneurysms. Recent reports also described the critical role of adventitial mast cells in the progression of atherosclerosis and aortic aneurysm [22]. Libby et al. [23], [24] provided new data on the importance of mast cells in atherosclerosis. In a manner similar to mast cells in the atherosclerotic intima and adventitia, the increased circulating basophil-mediated Th2-immune response, could play a role in the progression of atherosclerotic changes through the initiation of Th2, similar also to the circulating monocytes [15], [25] [26]. On the other hand, aerobic exercise is also reported to reduce the expression of Th2 cytokines in a mouse model [27]. Several groups have reported the inhibition of Th1 responses by statins [28]. Therefore, the combination therapy of aerobic exercise and statins could improve the imbalance in Th1/2 responses in atherosclerotic diseases by decreasing the number of basophils in peripheral blood. The present study is the first reports that demonstrated: (1) significant reduction in arterial wall stiffness and plasma basophil count after statin treatment combined with regular exercise intervention, and (2) a significant association between changes in arterial wall condition and plasma basophil count.

We investigated previously the use of atorvastatin and rosuvastatin combined with regular exercise on HDL-C in Japanese CAD patients [4]. Surprisingly, we found significant differences between the two statins; indicating the superiority of rosuvastatin, compared to atorvastatin, with exercise in increasing HDL-C and preservation of ubiquinol, an anti-oxidant effect. In the present study, we found (1) a significant decrease in arterial wall stiffness and plasma basophil count, and (2) a significant positive correlation between arterial wall stiffness and changes in plasma basophil count in the rosuvastatin group, but not in the atorvastatin group. It is probable that rosuvastatin inhibits the Th2-response by reducing basophil count, in addition to inhibiting the Th1 cytokines, and that the combination of exercise and rosuvastatin, compared to atorvastatin, has an anti-inflammatory and immuno-agonist properties in atherosclerotic lesions. Admittedly, the effect of rosuvastatin on the Th1/2 responses was not examined in detail in the present study. It has been shown that an increase in HDL-C and suppressed oxidative stress levels have a key role in the regression of the atherosclerotic changes [29], [30]. The increased HDL-C and the preserved levels of anti-oxidant molecule in rosuvastatin group, but not in atorvastatin, could be another possible mechanism for the amelioration of the arterial wall stiffness [4]. Little is known about why the difference existed according to two types of statin used, hydrophilic or lipophlic, on atherosclerosis. Several papers have reported the different effect of rosuvastatin and atorvastatin on atherosclerotic changes; ie, the greater decrease in coronary atheroma volume was observed in the rosuvastatin than atorvastatin group in CAD patients [31] and the different effects of Rho/Rho (ROCK) kinase pathway on the blood leukocytes [32]. These different several pathways on the vasculature and blood leukocytes might also be the possible cause of the different effects of these two statins on the arterial wall stiffness in the present study. Further large clinical trials are required to confirm the effects of rosuvastatin in arterial wall stiffness and circulating basophils.

Our study has several limitations. Firstly, the study did not distinguish between the separate effects of regular exercise and statin treatment; however, such a study would be ethically inappropriate for CAD patients. We also could not compare the subjects with or without the previous statin therapies before exercise intervention because all patients have not been treated by any statins and exercise training at the study entry. Secondly, the study included a small number of patients from a single center; therefore, the reproducibility of baPWV is of concern because of this small number of subjects. Yamashina et al. though have reported the high reproducibility and accuracy of baPWV in a small number of subjects [33]. Thirdly, baPWV is inaccurate in patients with peripheral artery disease, but there were no patients with peripheral artery disease in the present study. Fourthly, the validity and accuracy of automatic basophil measurement was not proven in the present study. It has been reported though to have a good correlation with basophil counts when utilizing XE-2100 and flow cytometric methods, in which CD123 and CD193 are used as basophil markers [34]. Fifthly, the supervised exercise training at the hospital was provided only once weekly although regular exercise are recommended 3 or more times weekly. As previously mentioned in the details [4], all patients were instructed on carrying out daily home exercise and brought their personal healthcare log, in which they recorded their exercise performance at home during the study. The patients who could not accomplish the home exercise despite the exercise instructor's advice were re-instructed on the proper way to perform the home exercise. Lastly, the blood levels of major Th1 and Th2 cytokines should have been measured to clarify the scientific rational behind the specific comparison between the reduced basophil counts and the improvement of arterial wall stiffness; however, it was difficult to examine additional molecules because this study was a sub-analysis and remaining blood samples are practically insufficient.

In conclusion, the present study demonstrated that the combination of statins and exercise therapy has significant vascular benefits probably by suppressing the inflammatory process in the arterial wall, and that the combination therapy correlated with reduction in circulating basophils in CAD patients with arterial wall stiffness.
