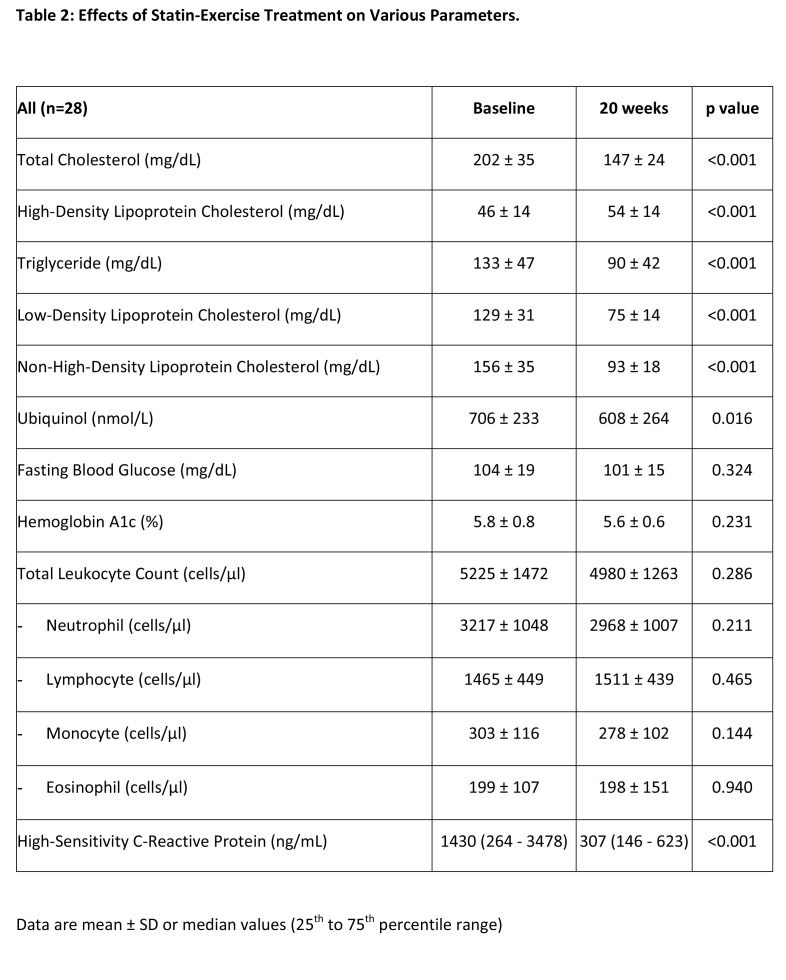# Correction: Combination Treatment of Rosuvastatin or Atorvastatin, with Regular Exercise Improves Arterial Wall Stiffness in Patients with Coronary Artery Disease

**DOI:** 10.1371/annotation/83ad6bd2-c729-47b3-87f9-6994b428eada

**Published:** 2013-01-17

**Authors:** Kensuke Toyama, Seigo Sugiyama, Hideki Oka, Yuri Iwasaki, Hitoshi Sumida, Tomoko Tanaka, Shinji Tayama, Hideaki Jinnouchi, Hisao Ogawa

In Table 2, the baseline data for High-Sensitivity C-Reactive Protein (ng/mL) was incorrect. The correct data is "1430 (264-3478)." Please see the corrected Table 2 here: 

**Figure:**